# A Rapid Assessment of Health Literacy and Health Status of Rohingya Refugees Living in Cox’s Bazar, Bangladesh Following the August 2017 Exodus from Myanmar: A Cross-Sectional Study

**DOI:** 10.3390/tropicalmed5030110

**Published:** 2020-07-01

**Authors:** Md Ridwanur Rahman, Mohammad Abul Faiz, Ma Yin Nu, Md Rafiqul Hassan, Ashish Kumar Chakrabarty, Iqbal Kabir, Khaleda Islam, Abul Kashem Mohammad Jafarullah, Mariam Alakabawy, Ameneh Khatami, Harunor Rashid

**Affiliations:** 1 Universal Medical College, Research Center, Dhaka 1212, Bangladesh; ridwanurr@yahoo.com (M.R.R.); forhadfahad@yahoo.com (A.K.C.); 2 Dev Care Foundation, Dhaka 1209, Bangladesh; drmafaiz@gmail.com; 3 Cox’s Bazar Medical College, Cox’s Bazar 4700, Bangladesh; drmayinnu@yahoo.com (M.Y.N.); mdrafiqulhasan28@gmail.com (M.R.H.); 4 National Institute for Preventive and Social Medicine, Dhaka 1212, Bangladesh; iqbalkabirdr@gmail.com; 5 Directorate General of Health Services (DGHS), Dhaka 1212, Bangladesh; akmjafar@gmail.com; 6 Graduate Programs in Public Health, University of New England, Portland, ME 04103, USA; malakabawy@une.edu; 7 Discipline of Child and Adolescent Health, Faculty of Medicine and Health, The University of Sydney, Westmead, NSW 2145, Australia; ameneh.khatami@health.nsw.gov.au (A.K.); harunor.rashid@health.nsw.gov.au (H.R.); 8 Department of Infectious Diseases and Microbiology, The Children’s Hospital at Westmead, Westmead, NSW 2145, Australia; 9 National Centre for Immunisation Research and Surveillance (NCIRS), The Children’s Hospital at Westmead, Westmead, NSW 2145, Australia

**Keywords:** Bangladesh, health literacy, health status, Myanmar, Rakhine, refugee, Rohingya

## Abstract

Background: A survey was conducted among Rohingya refugees to assess their overall health literacy and health status. Methods: A questionnaire was developed to conduct face to face interviews among Rohingya refugees in Cox’s Bazar, Bangladesh in November–December 2017. Families were selected using convenience sampling from four large refugee camps. Results: Primary respondents aged 10–90 (median 32) years, 56% male, representing 1634 families were interviewed and provided data of themselves and 6268 additional family members, 4163 (66.4%) of whom were children aged <18 years. Of all, only 736 (45%) primary respondents knew how to appropriately treat diarrhoea, 882 (54%) relied on unqualified village “doctors” for treatment, 547 (33.5%) reported a family member suffering injuries in the previous six months, with 8% (42/547) of injuries fatal. One hundred and ninety two (11.8%) primary respondents also reported deaths within their family in the preceding 12 months, with the majority (70% [134/192]) occurring in males, and 44% (85/192) of all deaths were claimed to be homicidal. Conclusion: This survey highlights overall poor health literacy, limited access to qualified health care, and a high rate of injuries and assaults among Rohingyas. However, these data come from an anecdotal survey that excluded some sensitive but important questions.

## 1. Introduction

The Rohingya people of Rakhine, Myanmar are considered one of the most persecuted populations in the world [1]. Rohingyas constitute about a third (now a quarter) of the population of Rakhine state (formerly known as Arakan), a western coastal state of Myanmar spreading over 36,760 square kilometres of land, with a population of about 3.2 million. Of the five districts of Rakhine, most Rohingyas are concentrated in Maungdaw. They are denied citizenship in Myanmar, which leads to negative discrimination, including denial of access to health and education. Rohingyas are also denied legal identities including birth certificates, and access to essential childhood vaccinations with 62% of Rohingya children under two receiving no parenteral vaccines [2]. Military crackdowns targeting Rohingyas have also occurred periodically, notably in 1978, 1991–1992, and most recently in 2017–2018, when approximately 700,000 Rohingyas crossed over the border to Bangladesh following escalating violence in Rakhine state, joining more than 200,000 Rohingya who were already in the country. Most of them now live in refugee camps in Cox’s Bazar, a coastal district of Bangladesh [1,3,4,5,6]. From the very beginning, a number of United Nations (UN) agencies, including United Nations High Commissioner for Refugees, World Health Organization (WHO), International Organization for Migration, United Nations Children’s Fund, and United Nations Population Fund; international humanitarian organisations including the International Federation of Red Cross and Red Crescent Societies, Médecins Sans Frontiers (MSF), CARE International, Save the Children Fund, and Orbis Eye Care; local non-government organisations including BRAC, Mukti, HOPE Foundation for Women and Children of Bangladesh, and Al-Markazul Islami are providing much needed humanitarian help. The Ministry of Health and Family Welfare, Bangladesh oversees and streamlines medical activities. There are medical clinics and dispensaries with facilities for minor surgeries within camps, and some over the counter drugs are available from shops and groceries around the camps accessible to both local residents and refugees. Patients needing secondary and tertiary care are transferred to local government medical college hospitals in Cox’s Bazar or Chittagong. All treatment, medications and diagnostic tests are free for the refugees. Traditional healers and traditional birth attendants may be active in the camps, but they are not easily identified or recognised outside of the small communities in which they practice. Previous studies have noted high rates of malnutrition and low immunisation coverage among Rohingya refugees in Cox’s Bazar who, thus, remain susceptible to infections including gastroenteritis, acute respiratory infections and acute jaundice syndromes [7,8,9,10,11]. Following the last mass migration, a large diphtheria outbreak and varicella and measles outbreaks have occurred, and cholera remains a constant threat [12,13,14,15]. Furthermore, Rohingya refugees suffer from a wide range of acute and chronic health conditions [7,14], including musculoskeletal and mental health problems that can be difficult to detect, assess and manage in this vulnerable population [16,17,18].

There has been some research to try to understand the magnitude of violence and fatalities occurring among Rohingyas [2,10,19]. A survey conducted among over 600 village leaders identified the primary reason for leaving Myanmar was violence in their village or in an adjacent village, perpetrated mostly by border police and the Myanmar military [19]. Another cluster of surveys led by MSF calculated the crude mortality rate (CMR) among those aged ≥50 years during the 2017 violence period was as high as 17.3 per 10,000 per day, an almost 15-fold higher CMR than in the same population before, and 9-fold higher CMR than after, the period of violence [10]. This is further corroborated by an in-depth interview of 22 survivors of a village called ‘Chut Pyin’, where an estimated 400 people with 99 children were killed in one day [20].

However, a comprehensive picture of the health status and health literacy (i.e., personal characteristics and social resources required for individuals and communities to access, understand and use information and services to make health decisions [21]) and other health care-related experiences among Rohingya refugees does not exist with respect to the most recent mass migration into Bangladesh. To this end, a rapid needs assessment survey was conducted among Rohingya refugees in Cox’s Bazar in late 2017 as a first step to inform strategies to provide adequate health care, resource mobilisation and develop further action plans for this vulnerable population.

## 2. Materials and Methods

A brief proposal was prepared outlining key study steps and submitted to the Ministry of Health and Family Welfare, Bangladesh and received formal approval. In consultation with researchers experienced in refugee health, a questionnaire was devised using the WHO Europe’s ‘Toolkit for assessing health system capacity to manage large influxes of refugees, asylum-seekers and migrants’ as a key reference. The questionnaire included questions on Rohingyas’ demographics, health literacy about symptoms and prevention of common illnesses, access to health care, sanitation and immunisations, current illnesses, injuries in the preceding six months and fatalities and animal bites encountered in the past one year, as well as the presence of disabilities at the time of the survey. The questionnaire was written in the local dialect using vocabulary predominantly used by lay Rohingya people with little or no literacy.

The survey was conducted with the help of 19 trained interviewers in four refugee camps in Cox’s Bazar from 25th November to 4th December 2017. Selection criteria for interviewers included an education level of at least 12th grade, ability to speak the local dialect (‘Rohain’ subdialect of Chittagonian Bangla language spoken in Cox’s Bazar), experience in conducting public health surveys, and successful completion of a training workshop and post-workshop assessment. A three-day structured training workshop (22–24th November 2017) was arranged by experienced researchers who have previously conducted large health surveys in resource-poor settings to train potential interviewers on various aspects of data collection, including how to obtain consent, how and where to check for a Bacillus Calmette–Guérin (BCG) vaccination scar, how to record and store data, and maintain confidentiality. An important focus of this workshop was on the ethical conduct of research including key aspects of good clinical practice (GCP) and the International Council for Harmonisation (ICH) guidelines and the necessity to comply with those principles. Of the 21 attendees, 19 successfully completed the formal assessment which included conducting a mock survey. The field work and data collection were supervised by two medically qualified experienced researchers. A debrief session was conducted during a study closure meeting on 4th December.

**Figure 1:**
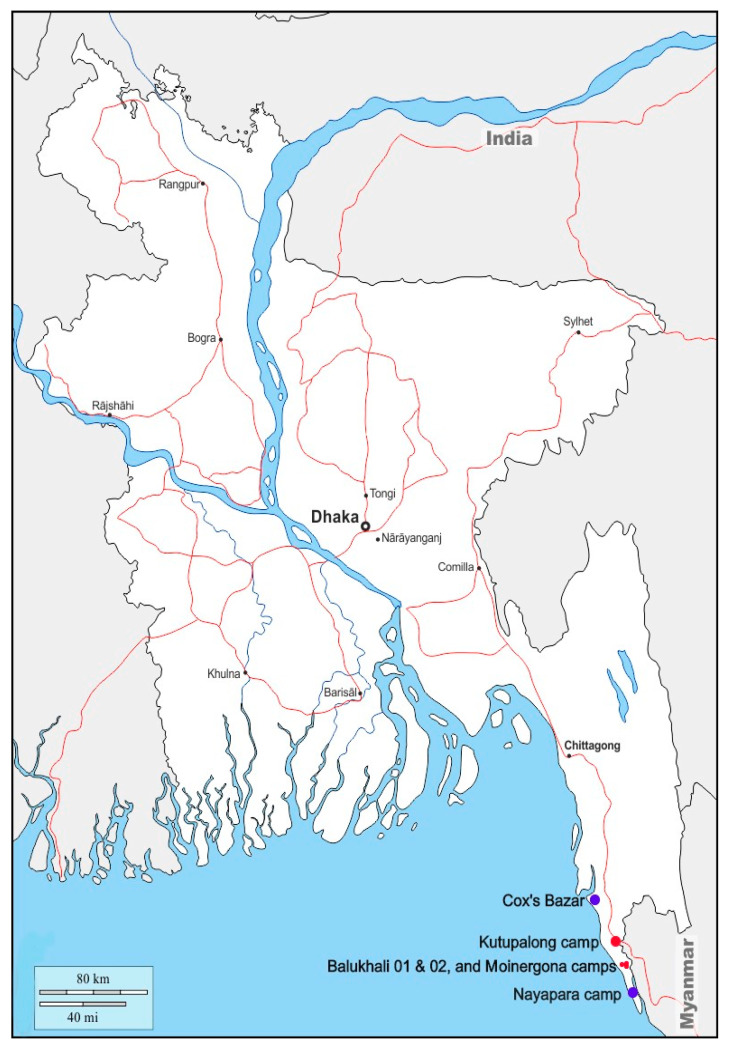
Map of Bangladesh showing Cox’s Bazar and study camps in red dots (source: https://d-maps.com).

Using a non-probability sampling method, consecutive houses from four refugee camps were surveyed starting from a corner of each camp which was chosen randomly. The camps where the survey was conducted are Balukhali Camp 01, Balukhali Camp 02, Moinergona Camp and Kutupalong Camp (Figure 1).

The interviewers approached the lead members (henceforth, called ‘primary respondents’) of the family and after explaining the survey aim and design, conducted a face to face interview to complete the questionnaire. Verbal agreement to participate in the survey and providing responses to interviewers’ questions were considered implied consent to participate in the study. Participants’ or their family members’ identifiable information were not collected, but age was. For the purposes of the survey, a ‘family’ was defined as a group of people who sleep under the same roof and share meals from the same pot. All data were entered on a master Microsoft Excel spread sheet before importing to Statistical Package for Social Sciences (SPSS) software (IBM SPSS Statistics for Windows, version 25.0, Armonk, NY: IBM Corp). Categorical data were expressed as number and proportion, while continuous data were expressed as range with measures of central tendency.

No formal sample size calculation was attempted for this survey. The initial study proposal aimed to recruit 800–1200 refugee families, but as the number of refugees over the weeks escalated, the recruitment aim was increased to about 1500 families. Although no sample size calculation was done, the aim was to capture data from about 1% of the refugees who migrated to Bangladesh in late 2017 (n = 700,000), that is about 7000 individuals. It was estimated that there would be an average of five people in each family, requiring interviews of key informants from about 1500 families. This sample estimate was inflated by 10% to account for any incomplete data.

In 2017, following a large influx of Rohingyas into Bangladesh, the Directorate General of Health Services (DGHS), MHFW, Bangladesh approved immediate commencement of the study without prior ethics approval (Ref: DGHS/PHC/Rohingya/2017/163) as understanding the refugees’ health status and risks was considered critically important for the refugees themselves and for the host population. The study was conducted in compliance with the ICH and GCP guidelines. All key investigators were qualified clinical research professionals, including a WHO Monitor (M.R.R.), and the interviewers were assessed to ensure their understanding of ethical principles before being sent to the field. Verbal consent from each interviewee was obtained following detailed explanation of the survey methodology, including its purpose and its voluntary nature and explaining the participants’ right to leave the interview at any time. The data were stored and managed confidentially and no one other than the investigators or their authorised personnel had access to the data. Children who provided data did so under supervision of their adult family members.

## 3. Results

### 3.1. Demographics

The demographics of primary respondents, their access to health care and their economic background are summarised in Table 1 and Table 2, and the demographics of their family members are summarised in Table 3.

A total of 1634 primary respondents were approached, and all agreed to participate in the survey and provided data on an additional 6268 family members. About 97% of primary respondents (1582/1634) hailed from Maungdaw township, the rest 3.2% (52/1634) were from other places including Buthidaung, Pauktaw, Rathedaung and Taungup. Most had no or limited literacy (75% of primary respondents and 86% of family members had, at most, five years of education). Some sort of employment was documented in 57% of primary respondents (927/1634), but only 18% of other family members (1108/6268). Excluding children aged <18 years (n = 4272) in the whole cohort, approximately 56.1% (2035/3630) of individuals for whom information was available were employed. The range of responses to income and ownership of land and gold varied widely, but median values were low. The median monthly income for primary respondents was US$65 per month.

### 3.2. Health Literacy and Health Status

Primary respondents’ health awareness and access to health care are summarised in Table 4. Overall, there was poor understanding regarding treatment of common illnesses such as diarrhea. The majority (90%) of childbirths occurred at home, with only 4% occurring in the presence of a trained health care worker. Data regarding injuries or animal bites suffered within the preceding six months, presence of ongoing disabilities and fatalities occurring in the last year among all family members are summarised in Table 5. Over a third (547/1634) of primary respondents reported injuries among themselves or their family members in the previous six months, and over one-eighth (192/1634) reported deaths among family members in the previous 12 months. At the time of the interview, 24.7% (403/1634) of primary respondents reported to have an illness, and only 62.1% (1015/1634) managed to have some sleep the previous night.

## 4. Discussion

Key findings of the survey include overall poor health literacy, limited access to health care, including primary and preventive care, as well as obstetric care, and a high rate of injuries and assaults. This survey was conducted in November–December 2017, three months after the start of the most recent violence in Rakhine state, Myanmar, giving an overall picture of the health status and health literacy of the Rohingya refugees that fled to Bangladesh and were temporarily settled in Cox’s Bazar. The timing of the survey and the questions that were asked, generally assessing health parameters over the preceding 12 months, reflect the status of this population prior to their migration and provide important information regarding health needs for service providers in Bangladesh.

The demographic characteristics of the respondents in this survey demonstrate the basic existence with which most Rohingyas live, such as living in thatched or leave-roofed houses in the vast majority, with a median monthly income of US$ 65, which is just above the World Bank definition of absolute poverty set at US$ 1.90/day in 2015 [22]. This is in line with previous reports assessing the socioeconomic status of this marginalised population [2]. Although access to health care is multifactorial and complex, economic factors play a key role [23]. As such, over 80% of Rohingyas, while in Rakhine, predominantly relied on traditional village “doctors” or “pharmacists” for their medical care. Such traditional healers play an important role in the health and well-being of many marginalised and vulnerable populations, and ongoing constructive dialogue between traditional health providers and formally recognised medical services is essential to ensure all health and mental health needs of communities in need are met [24]. Access to antenatal and obstetric care was also limited. Almost two-thirds of pregnant women did not receive any antenatal care and 90% relied on domiciliary care by unregulated or unqualified health care providers for their deliveries. Most of these findings confirm those published previously on this subject [2,10,19]; however, this study also uniquely identifies that Rohingyas have poor health literacy, with over half of primary respondents unable to answer questions on how to appropriately treat diarrhoea. This is in the context of a setting in which diarrhoea is endemic, and a leading cause of death [25].

However, there were other positive findings from the survey. Over 80% of families had access to a tube well (an iron pipe well meant for suctioning water from underground aquifers) for water, which has also been reported by other researchers [2], and 97% had at least one sanitary latrine for the family; although, hand washing with soap was suboptimal, with only two-thirds of primary respondents reporting use of soap and water to wash hands after going to the toilet. Despite this seemingly low rate of basic infection prevention, this is an improvement compared to a previous survey among community members in rural Myanmar conducted in the 1980s, which found that only 5% to 12% of people regularly used soap to wash their hands after visiting the toilet [26]. However, this should be considered against the real-life context that the vast majority of Rohingyas were struggling hard to make ends meet and were forced to choose between the purchase of soap and the most basic essentials such as food.

Around 84% of primary respondents reported ever receiving a vaccine, and a BCG scar was noted in 60% of children among all family members under five years of age. No further information regarding vaccinations received was sought and the reports were not corroborated by viewing vaccination certificates, which were unlikely to be available. This is because one of the aims of this survey was to crudely gauge the refugees’ prior access to preventive medicine rather than establishing a full immunisation record. Unfortunately, this suggests that up to 16% of individuals may not have received any immunisations in the past, including those in the WHO Expanded Program on Immunisation schedule, leading to a significant risk of both individual and community vulnerability to disease outbreaks as have occurred with diphtheria, measles and varicella [12,13,15]. However, our results are more favourable than those of other recent surveys that demonstrated 43% of children under the age of four had not received any doses of an injectable vaccine in Myanmar [2], and that only 23% of Rohingya children under five years of age had received a measles vaccine [10]. Explanations for these differences are multifactorial and discussed below.

Another important finding from this survey is the relatively large proportion of Rohingyas who had suffered injuries within a six-month period. Among injuries reported, the largest proportion were those due to assault, including by stick (46.4%), bullet (23.6%) and knife (5.8%). This gives a crude estimate of the assault rate in this population, excluding homicides, as approximately 2417 per 100,000 persons within the previous year, significantly higher than the background rate of non-fatal assaults occurring in Myanmar of 8.7 cases per 100,000 persons in 2016 [27]. This survey did not ask for further information regarding how injuries occurred, and by whom assaults were perpetrated; however, in a separate survey conducted subsequent to ours, 64% of respondents reported violence against civilians occurring during the military campaigns in Rakhine by Myanmar security forces during August–September 2017 [19]. In addition, almost two-thirds of injuries reported in the current survey led to death or ongoing complaint or disability, reflecting the severity of the injuries.

Fatalities were common, with 192 deaths occurring among all family members over the preceding year, with the most common cause being homicide (44%), although illness and accidents were also responsible for many of those deaths. This high number of fatalities corroborate other estimates that suggest around 6700 Rohingyas died as a result of violence in the initial 31 days following the outbreak of unrest [10,28]. Similarly, Bhatia et al. record 10.7% of Rohingya families surveyed reported one death in the family, 2.5% reported two deaths, and 1.2% reported three deaths in the one year preceding the survey [2]. In the current survey, victims of fatalities were predominantly male (approximately 70%), consistent with the findings of another survey conducted by MSF [10]; however, in contrast to their results, we note more people aged 50 years or younger dying compared to those aged over 50 years. This difference could be explained by the temporal relation to the commencement of violence in Rakhine, wherein our study took place within three months of the outbreak of violence, while the MSF study began in February 2017, six months before the 2017 violence, although it continued until November 2017. Sadly, eight of the fatalities (4%) reported here were maternal deaths indicative of poor/non-access to adequate obstetric and perinatal care for Rohingyas [1].

In addition, 48 primary respondents reported a snake bite in a family member in the preceding year. This roughly translates to a snake bite incidence in this population of 0.6% compared to 0.12% incidence in central Myanmar; although generally, 1 in 15 snake bites in Myanmar are fatal [29], and no fatal snake bites were reported in this survey. Dog bites among families were reported by 104 primary respondents, with five (5%) of these resulting in death. This may be related to rabies infection, which is still a major public health concern in Asia, including Myanmar. The 2015 estimated rate of rabies mortality across Myanmar was 0.2 per 100,000 population, and the rate is claimed to be lower in Rakhine [30]. These five fatal dog bites among a cohort of 7900 people translates to a mortality rate of 63 per 100,000 persons. Although some proportion of these deaths may have been due to blood loss, organ damage, wound infection or other causes, rather than rabies, it is likely that the poor public health infrastructure in Rakhine results in an under-reporting and under-estimation of rabies deaths among Rohingyas.

Despite the generally young age of the population surveyed (median age of primary respondents was 32 years, and of their family members was 12 years with 71% [4477/6268] of family members aged ≤18 years), around 25% of primary respondents and 12% of their family members reported illness at the time of the survey, and just over 60% of primary respondents managed to get sleep in the preceding night. These questions again only broadly assess ongoing stress and mental health issues among the refugees and highlight the large un-met need to access health care, including mental health care. A cross-sectional study conducted among existing Rohingya refugees in Bangladesh before the 2017 exodus showed that 36% suffered from post-traumatic stress disorder (PTSD) and 89% suffered from depression [16]. Unsurprisingly, high rates of mental health problems were also common among children, with 52% of Rohingya children in Bangladesh having results in the abnormal range for emotional symptoms on the Strengths and Difficulties Questionnaire (SDQ), and 25% with results in the abnormal range for peer problems [31]. Rohingya refugees in Malaysia have similar high rates of comorbid mental health disorders, including 32% with PTSD, 9% with generalised anxiety disorders, and 12% with major depressive disorder [32].

Such high rates of illness and mental health problems in these refugees suggest ongoing vulnerability to disease that is much higher than would be expected by the population demographics. For example, recently, 38 COVID-19 cases with two fatalities have been reported among Rohingya refugees, including among residents of our study camps, which is a grave concern due to the crowded and difficult social circumstances within these camps [33]. Lockdown measures have been introduced following the detection of early cases, and surveillance continues. In addition to monitoring the direct health and mental health impact of the pandemic in these camps, assessing both the efficacy of lockdown measures, as well as negative effects on access to health care and other services will be crucial in the coming months.

The strength of this study is its large sample size, which was selected in a systematic manner from four large refugee camps, and which is probably representative of the population of Rohingya refugees currently displaced to Cox’s Bazar in Bangladesh. The study was conducted within three months of the influx of refugees and the researchers had field experience in this context and were familiar with the local culture and language.

There are several limitations to the study. Firstly, the anecdotal nature of the survey, and the interviewers’ inability to objectively corroborate statements (with the exception of BCG scars in children), means that both under- and over-estimations are highly likely and only general conclusions can be drawn. Secondly, some primary respondents were children aged as young as 10 years, although children aged 10 to <18 years only accounted for 0.6% of all primary respondents. Thirdly, for cultural and political reasons, we did not ask for details regarding injuries and fatalities, in particular, the perpetrators of injuries and fatalities. Thus, although we postulate, based on the timing of the majority of deaths that occurred in the preceding four months, that a large proportion resulted from the violence occurring in Rakhine, we cannot confidently support or refute this. Finally, it is likely that despite efforts to ensure the surveys were conducted in a language and culture-sensitive manner, a degree of misunderstanding occurred. For example, the question regarding receipt of a vaccine may have been interpreted as having received a vaccine after arriving in Bangladesh, since several vaccination campaigns were rolled out to curb epidemics of diphtheria, measles, varicella and cholera [34]. Thus, it is possible that the routine immunisation rate in this population is higher than reported in this survey; although, based on the results of other similar surveys referred to earlier, it is unlikely that the under-estimation is large. Similarly, some data conflict with the results of other surveys. For example, in our survey, only 24% of primary respondents and 33% of their family members were reported as not having any education, while in another survey, 76% of Rohingya household members older than 15 years had no formal education, and 53% of Rohingya children aged younger than 15 years did not attend school [2]. This could be due to a failure of the survey in distinguishing formal and informal education, such as that provided by religious and village leaders. Finally, the data cannot be broadly generalised as they are drawn from only 1634 families representing just 1% of the Rohingya influx and were recruited using convenience sampling.

## 5. Conclusions

This survey provides a broad-strokes overview of Rohingya refugees’ health status and health literacy and highlights overall poor health literacy and limited access to qualified health care in Myanmar. A high rate of injuries, accidents and assaults, as well as fatalities, have occurred in this population in the preceding 12 months, with the majority of deaths occurring in the preceding four months, coinciding with the outbreak of violence in Rakhine state in Myanmar that lead to the mass migration of Rohingyas into Bangladesh. Furthermore, despite the generally young age of the population surveyed, there appears to be a high rate of ongoing or persistent illness and disability, reflecting the multifactorial trauma and socioeconomic disadvantage experienced by these individuals. These findings make timely and multi-pronged health, educational and political interventions imperative to ensure the physical, mental, social and spiritual wellbeing of this vulnerable population.

## Figures and Tables

**Table 1:** Demographic characteristics of primary respondents among Rohingya refugees.

Particulars (N = 1634)	Overall n (%)	Male n (%)	Female n (%)	*p* Value
**Number of participants**	1634	913 (55.9)	721 (44.1)	
**Age in years (median, mean ± SD)**	10–90 (32, 36.3 ± 13.8)	10–90 (35, 37.7 ± 14.6)	10–80 (30, 34.4 ± 12.5)	<0.01
**Children (aged < 18 years)**	9 (0.6)	7 (0.8)	2 (0.3)	0.19
**Occupation in Myanmar**				
**Farmer**	423 (25.9)	393 (43.0)	30 (4.2)	<0.01
**Homemaker**	629 (38.5)	5 (0.5)	624 (86.5)	<0.01
**Grocery businessman**	211 (12.9)	199 (21.8)	12 (1.7)	<0.01
**Sedentary workers**	58 (3.5)	56 (6.1)	2 (0.3)	<0.01
**Fisherman**	32 (2)	32 (3.5)	0 (0)	<0.01
**Student**	32 (2)	24 (2.6)	8 (1.1)	0.03
**Labourer**	108 (6.6)	97 (10.6)	11 (1.5)	<0.01
**Others**	95 (5.8)	77 (8.4)	18 (2.5)	<0.01
**Retired**	32 (2.0)	25 (2.7)	7 (0.8)	0.01
**Unemployed**	14 (0.9)	5 (0.5)	9 (1.2)	0.13
**Years of education received**				
**No education**	389 (23.8)	241 (26.4)	148 (20.5)	0.01
**1–5 years**	834 (51.1)	403 (44.1)	431 (59.8)	<0.01
**6–10 years**	340 (20.7)	208 (22.8)	132 (18.3)	0.03
**11–12 years**	51 (3.2)	41 (4.5)	10 (1.4)	<0.01
**>12 years**	20 (1.3)	20 (2.2)	0 (0)	<0.01

SD = Standard deviation.

**Table 2:** Economic background of primary respondents among Rohingya refugees.

Particulars (N = 1634)	Overall n (%)
**Owned lands in Rakhine**	1278 (78.2)
**Total arable land in acres, range (median; IQR)**	0.4–144 (2; 0.8–4.0)
**Own gold/jewelleries**	1310 (80.2)
**Total amount of gold in grams, range (median)**	1–478 (23.3)
**Family income per month in US$ before migration, range (median; IQR)**	0–5200 (65; 65–195)
**Have money deposited in a bank**	12 (0.7)
**Roof of your Myanmar house built with**	
**Leaves**	909 (55.6)
**Thatched**	597 (36.5)
**Corrugated iron sheets**	111 (6.8)
**Others**	17 (1)
**At home, where did you usually get your drinking water from?**	
**Tube well**	1316 (80.5)
**Pond**	174 (10.6)
**Deep well**	105 (6.4)
**Other sources**	39 (2.4)
**Have sanitary latrine for the family in Myanmar**	1583 (96.9)
**One latrine**	1267 (77.5)
**More than one latrine**	316 (19.3)

IQR = Interquartile range.

**Table 3:** Demographic characteristics of family members among Rohingya refugees.

Particulars	Overall n (%)	Male n (%)	Female n (%)	*p* Value
**Total number of participants**	6268	2973 (47.4)	3295 (52.6)	
**Age in years range (median, mean ± SD)**	0.1–120 (12, 15.9 ± 14.6)	0.1–98 (11, 15.1 ± 14.7)	0.1–120 (13, 16.5 ± 14.4)	<0.01
**Children (aged < 18 years)**	4163 (66.4)	2114 (71.1)	2049 (62.2)	<0.01
**Occupation in Myanmar**				
**Student**	2217 (35.4)	1129 (38.0)	1088 (33.0)	<0.01
**Homemaker**	1111 (17.7)	19 (0.6)	1092 (33.1)	<0.01
**Farmer**	363 (5.8)	339 (11.4)	24 (0.7)	<0.01
**Grocery businessman**	198 (3.2)	189 (6.4)	9 (0.3)	<0.01
**Labourer**	150 (2.4)	145 (4.9)	5 (0.2)	<0.01
**Others**	334 (5.3)	182 (6.1)	152 (4.6)	0.01
**Unemployed or too young to be employed**	1832 (29.2)	940 (31.6)	892 (27.1)	<0.01
**Retired**	63 (1)	30 (1)	33 (1)	0.98
**Years of education received**				
**No education**	2064 (32.9)	1057 (35.6)	1007 (30.6)	<0.01
**1–5 years**	3322 (52.9)	1434 (48.2)	1888 (57.3)	<0.01
**6–10 years**	810 (13)	423 (14.2)	387 (11.7)	<0.01
**11–12 years**	51 (0.8)	39 (1.3)	12 (0.4)	<0.01
**>12 years**	21 (0.3)	20 (0.7)	1 (0.03)	<0.01
**Ever received a vaccine**	5255 (83.8)	2475 (83.2)	2780 (84.4)	0.23
**BCG vaccination in children < 5 years (N = 1264)**	764 (60.4)	381 (12.8)	383 (11.6)	0.15
**Are they ill now? (Yes)**	778 (12.4)	318 ()	460 (14)	<0.01

SD = Standard deviation.

**Table 4:** Health literacy and access to health care among Rohingya refugees during their stay in Myanmar, as reported by primary respondents.

Questions	Number (%) (Total N = 1634)
**How do you treat if someone at home suffers from diarrhoea?**	
**With oral rehydration salt**	736 (45)
**With medicine**	247 (15.1)
**Other**	54 (3.3)
**No response provided**	597 (36.5)
**Do you wash your hands with soap after the toilet? (Yes)**	1092 (66.8)
**Where do you go first when a family member is ill?**	
**Unqualified village doctor**	882 (54)
**Pharmacy/dispensary**	449 (27.5)
**Government hospital**	274 (16.8)
**Other**	29 (1.7)
**Any babies born in the family in the last one year? (Yes)**	397 (24.3)
**Did a pregnant woman in your family ever receive a vaccine? (Yes)**	1102 (67.4)
**Did a pregnant woman in the family ever receive antenatal care? (Yes)**	562 (34.4)
**Place of delivery of the last baby born to the family**	
**At home**	1464 (89.6)
**In hospital**	63 (3.9)
**Other places**	107 (6.5)
**Who delivered (or helped deliver) the last baby born in the family?**	
**A traditional birth attendant**	1178 (72.1)
**A relative**	291 (17.8)
**A nurse/mid-wife or doctor**	71 (4.3)
**Other**	94 (5.8)

**Table 5:** Injuries, animal bites and deaths among Rohingya families as reported by primary respondents interviewed in Cox’s Bazar.

Particulars	Number (%)
**Any injury among family members in the last six months (N = 1634)? (Yes)**	547 (33.5)
**Injury type (N = 547)**	
**Assault**	286 (52.3)
**Accident**	128 (23.4)
**Occupational**	51 (9.3)
**Domestic task**	41 (7.5)
**Other**	41 (7.5)
**Assault caused by (N = 276)**	
**Stick**	128 (46.4)
**Bullet**	65 (23.6)
**Knife**	16 (5.8)
**Burn**	4 (1.4)
**Other**	63 (22.8)
**Treatment received (** **N = 547)**	
**From pharmacy**	211 (38.6)
**From hospital**	158 (28.9)
**From primary care centre**	47 (8.6)
**No treatment received**	108 (19.7)
**Consequence of injuries (N = 547)**	
**Complete resolution**	195 (35.6)
**Ongoing complaint or disability**	310 (56.7)
**Death**	42 (7.6)
**Any snake bites** **among family members in the last six months (N = 1634)? (Yes)**	48 (2.9)
**Fatalities from snake bites (N = 48)**	0 (0)
**Any dog bites** **among family members in the last six months (N = 1634)? (Yes)**	104 (6.3)
**Fatalities from dog bites (N = 104)**	5 (5%)
**At least one death among family members in the last one year (N = 1634)**	192 (11.8)
**Two deaths in the family**	25 (1.5)
**Three deaths in the family**	2 (0.1)
**Gender of deceased (N = 192)**	
**Male**	134 (69.8)
**Female**	54 (28.1)
**Unspecified**	4 (2.1)
**Age of deceased in years, range (median)**	0.1–113 (31)
**Deceased aged** **≤** **50 years**	116 (60.4)
**When did the individual die? (N = 192)**	
**Within the preceding 4 months**	114 (59.4)
**4–12 months prior**	78 (40.6)
**Cause of death (N = 192)**	
**Homicide**	85 (44.2)
**Sudden unexpected death**	34 (17.7)
**Febrile illness**	19 (9.9)
**Paralytic illness**	11 (5.7)
**Accident**	9 (4.7)
**Maternal death**	8 (4.2)
**Coma**	4 (2.1)
**Other**	18 (9.4)
**Unknown**	4 (2.1)
